# An expression system for screening of proteins for glycan and protein interactions

**DOI:** 10.1016/j.ab.2010.12.036

**Published:** 2011-04-15

**Authors:** Diana M.E. Otto, Maria A. Campanero-Rhodes, Rositsa Karamanska, Andrew K. Powell, Nicolai Bovin, Jeremy E. Turnbull, Robert A. Field, Jonathan Blackburn, Ten Feizi, Paul R. Crocker

**Affiliations:** a Division of Cell Biology and Immunology, Wellcome Trust Biocentre, College of Life Sciences, University of Dundee, Dundee DD1 5EH, UK; b Glycosciences Laboratory, Division of Medicine, Imperial College London, Northwick Park and St. Mark’s Campus, Harrow HA1 3UJ, UK; c Department of Biological Chemistry, John Innes Centre, Norwich NR4 7UH, UK; d School of Biological Sciences, University of Liverpool, Liverpool L69 7ZB, UK; e M.M. Shemyakin and Y.A. Ovchinnikov Institute of Bioorganic Chemistry, Russian Academy of Sciences, 117997 Moscow, Russian Federation; f Division of Medical Biochemistry, Institute for Infectious Disease and Molecular Medicine, Faculty of Health Sciences, University of Cape Town, Rondebosch, 7700 Cape Town, South Africa

**Keywords:** Cloning, Expression, Lectin, Glycan, Protein

## Abstract

Here we describe a versatile high-throughput expression system that permits genome-wide screening of type 1 membrane and secreted proteins for interactions with glycans and proteins using both cell-expressed and soluble forms of the expressed proteins. Based on Gateway cloning methodology, we have engineered a destination vector that directs expression of enhanced green fluorescent protein (EGFP)-tagged proteins at the cell surface via a glycosylphosphatidylinositol tail. The EGFP fusion proteins can then be cleaved with PreScission protease to release soluble forms of proteins that can be optionally biotinylated. We demonstrate the utility of this cloning and expression system for selected low-affinity membrane lectins from the siglec family of sialic acid-binding immunoglobulin-like lectins, for the glycosaminoglycan-binding proteins FGF-1 and BACE, and for the heterotypic adhesion molecules JAM-B and JAM-C. Cell-expressed proteins can be evaluated for glycan interactions using polyvalent soluble glycan probes and for protein interactions using either cells or soluble proteins. Following cleavage from the cell surface, proteins were complexed in solution and sufficient avidity was achieved to measure weak protein–glycan and weak protein–protein interactions using glycan arrays and surface plasmon resonance, respectively.

Cellular recognition by membrane and secreted proteins commonly involves both protein–protein and protein–glycan interactions, with the latter being mediated by lectin-like proteins [1]. Protein–glycan interactions are important in regulating a wide variety of physiological functions, especially in the nervous and immune systems, and their dysregulation can contribute significantly to human pathologies [2–4]. In mammals, a growing number of lectin-like receptors and secreted proteins are being identified, and many have been assigned to discrete families based on sequence similarity, including C-type lectins, galectins, and siglecs (sialic acid-binding immunoglobulin [Ig]-like lectins)^4^ [4,5]. It is likely that many other proteins mediate biologically important glycan recognition functions, but a systematic approach is required to screen membrane and secreted proteins for their ability to interact with glycans. The recent availability of publicly accessible mammalian gene collections [6] and the development of glycan array technologies [7–10] have opened up the possibility of systematically screening expressed proteins of interest against hundreds of different glycan structures and defining novel lectin-like activities of proteins.

Protein–glycan interactions are typically of low affinity and often depend on clustering of both the receptor and ligand to mediate high-avidity binding [11]. Protein expression strategies for glycan array screening need to take this into account. One approach is to express proteins as preformed multimers or to engineer tags that can be exploited for multimerization in solution by using appropriate cross-linking agents. Another strategy is to express candidates on the cell surface in a format that is permissive to ligand-induced clustering, resulting in high-avidity binding. Glycosylphosphatidylinositol (GPI) anchors are a useful way to achieve this because they are embedded in the outer leaflet of the plasma membrane and can localize within discrete membrane microdomains [12]. Because they are not tethered directly to cytoskeletal proteins, they are more likely to be freely diffusible and available for ligand-induced clustering [13]. Furthermore, GPI anchors are able to transport proteins efficiently to the cell surface [14].

Besides protein–glycan interactions, many biologically important protein–protein interactions can also be of low affinity, particularly those involved in transient and regulated interaction such as those at the cell surface, and this is a particular feature of the immune system where cellular contacts are often transient and regulated [15]. Therefore, the same system that is designed for analysis of low-affinity glycan interactions can also be used to screen for protein-dependent interactions.

Here we describe a Gateway-based cloning system [16] that allows high-throughput cloning and expression of secreted and type 1 membrane proteins that can be screened for glycan- and protein-binding properties. A particularly useful feature of this system is that tagged proteins are initially expressed as GPI-anchored membrane receptors that can then be cleaved selectively from the cell surface using human rhinovirus 3C protease (PreScission protease [PP]). This versatility allows proteins of interest to be screened for molecular interactions when presented either at the cell surface or in solution following cleavage. We demonstrate that this expression system can be used to measure weak protein–glycan and protein–protein interactions using several well-established membrane and secreted proteins, including siglecs, the heparan sulfate-binding proteins FGF-1 (fibroblast growth factor 1) and BACE (β-site APP [amyloid precursor protein]-cleaving enzyme), and a pair of heterotypic junctional adhesion molecules (JAMs), JAM-B and JAM-C. The broad flexibility of this high-throughput system can be applied to genome-wide investigations of protein–glycan and protein–protein interactions.

## Materials and methods

### Generation of destination vectors

The pEGFP–N1 cloning vector (BD Biosciences Clontech, GenBank Accession No. U55762) that encodes enhanced green fluorescent protein (EGFP), a red-shifted variant of the wild-type green fluorescent protein (GFP) gene, was linearized with blunt-end restriction enzyme SmaI. Ligations of vector with the Gateway C1 cassette, containing the suicide *ccdB* box with *ccdB* and chloramphenicol genes and *attR* sites (Invitrogen, Product No. 11828-019), were transformed into the DB3.1 *Escherichia coli* strain and positive clones were selected on chloramphenicol plates. Correct orientation of the cassette was verified by restriction digests and sequencing, resulting in the EGFP–pDEST vector. The GPI signal of the TRAIL receptor 3 was amplified by the polymerase chain reaction (PCR) using a complementary DNA (cDNA) clone kindly supplied by D. Legler [12]. PCR primer sequences used in this study are provided in Supplementary material. The PCR product was digested, purified, and subcloned into the BsrGI/NotI sites of EGFP–N1. The EGFP–GPI domain was digested with AgeI/DraIII and cloned into the EGFP–pDEST vector, resulting in EGFP–GPI–pDEST. A PP site [17] was added using a QuickChange site-directed mutagenesis kit (Stratagene) to generate an EGFP–PP–GPI–pDEST vector. A minimal 14-residue peptide tag (LNDIFEAQKIEWHE) called the Avitag [18], which can be biotinylated by BirA, was PCR amplified and cloned into the vector using the QuickChange site-directed mutagenesis kit together with a 7-residue linker (GSPGSPG) to generate the final destination vector EGFP–LNKAVI–PP–GPI–pDEST.

### Generation of entry and expression clones

The signal peptides and extracellular regions from selected cDNAs were amplified by PCR. The PCR samples were gel purified and subjected to the BP reaction using BP Clonase mix and the pDONOR207 entry vector (Invitrogen). BP Clonase is a proprietary enzyme formulation containing the bacteriophage lambda recombination protein Int (integrase) and the *E. coli*-encoded protein IHF (integration host factor) that catalyze the in vitro recombination of PCR products or DNA segments from clones (containing att**B** sites) and a donor vector (containing att**P** sites) to generate entry clones. Correct entry clones were identified by restriction digest analysis and further used in LR reactions (Invitrogen) with the EGFP–LNKAVI–PP–GPI–pDEST vector to generate expression clones. LR Clonase II enzyme mix contains a proprietary blend of Int, IHF, and Xis (excisionase) enzymes that catalyze the in vitro recombination between an entry clone (containing a gene of interest flanked by att**L** sites) and a destination vector (containing att**R** sites) to generate an expression clone. Restriction digests identified correct clones, which were further verified by sequencing. BP and LR reactions routinely showed at least 90% cloning efficiencies.

### Generation of stable cell lines

Chinese hamster ovary (CHO) cells (5 × 10^6^) were transfected with expression clones by electroporation in Ham’s F12 + 20% fetal calf serum (FCS), using a Bio-Rad electroporator and Bio-Rad 0.4-cm cuvettes, at 260 V, 960 °F, and 50 μg of DNA. Cells were cultured for 2 weeks in medium containing 1.0 mg/ml neomycin (G418 disulfide), and drug-resistant cells were sorted for EGFP expression using a FACS Vantage SE cell sorter (Becton Dickinson, Oxford, UK). Cell lines were sorted for a second or third time using biotin anti-EGFP antibody (Ab) (Vector Labs) and streptavidin–allophycocyanin (APC) staining (BD Biosciences).

### Production of biotinylated soluble recombinant protein

CHO cells were grown as adherent cultures and harvested by incubation for 10–30 min in phosphate-buffered saline (PBS) containing 10 mM ethylenediaminetetraacetic acid (EDTA) at 37 °C. After washing, 6 × 10^7^ cells were suspended in 300 μl of Ca^2+^- and Mg^2+^-free Hank’s buffered salt solution containing 0.5 mM EDTA and 1 mM dithiothreitol. PP was added at a final concentration of 15 μg/ml. The cell suspension was incubated at 4 °C for 4 h. The cells were spun down, and the PP digestion was stopped by the addition of the sulfhydryl blocking agent iodoacetamide (10 μM). Recombinant proteins were analyzed by Western blotting using 5–15% sodium dodecyl sulfate–polyacrylamide gel electrophoresis (SDS–PAGE) linear gradient polyacrylamide gels (Novagen) followed by transfer to nitrocellulose membranes (Schleicher–Schuell). Blots were blocked in casein solution and incubated with biotin goat anti-GFP Ab (10 μg/ml, Vector Labs) followed by a preformed complex between streptavidin and biotinylated alkaline phosphatase (Vectastain ABC–Amp, Vector Labs). The chromogenic substrate for alkaline phosphatase, 5-bromo-4-chloro-3-indolyl phosphate/nitroblue tetrazolium, was provided by the DuoLuX detection kit (Vector Labs). Protein concentrations were estimated by comparing the intensity of protein bands at two dilutions with that obtained with a GFP standard that was routinely included at 1 and 3 ng on each blot.

Biotinylation was performed with BirA according to the manufacturer’s guidelines (Avidity, Denver, CO, USA). Protein (250 ng) was incubated in 50 mM d-biotin and 10,000 U of BirA at room temperature for 1.5 h in a volume of 300 μl. Excess biotin was removed by desalting into PBS using fast ultrafiltration filter devices (Millipore, Consett, UK). Biotin incorporation was analyzed by Western blotting as described above except that the biotin anti-GFP step was omitted.

### Cellular binding assays

Cell binding to specific glycans attached to biotinylated polyacrylamide (PAA) was determined as described previously [19]. Briefly, cells were detached from tissue culture flasks using PBS containing 10 mM EDTA at 37 °C and treated with *Vibrio cholerae* sialidase (0.05 U/ml) at 37 °C for 1 h to unmask siglecs as described before [19]. Cells were then incubated with biotinylated PAA compounds [20] at 20 μg/ml for 1 h on ice followed by streptavidin–APC (0.5 μg/ml) for 30 min prior to analysis on a FACSCalibur system (BD Biosciences).

Cell-to-cell binding mediated by JAM-B and JAM-C was assessed using a flow cytometric assay in which CHO cells expressing each adhesion molecule were differentially labeled with fluorescent markers and the presence of cell couples was detected by dual fluorescence. Differential labeling was achieved using the lipophilic carbocyanine dyes DiI and DiD (Invitrogen), which emit light at 565 and 665 nm, respectively. Following labeling according to the manufacturer’s guidelines, cells were washed, mixed together in 1:1 ratio, and pelleted by centrifugation, and the pellets were gently mixed and then incubated on ice for 1 h. After gentle resuspension, the extent of cell–cell binding was determined using a FACSCalibur.

Cell binding to recombinant proteins was performed by incubating 0.5 × 10^5^ wild-type or transfected CHO cells with 0.8 μg/ml biotinylated proteins for 1 h on ice. After washing, cells were incubated with streptavidin–APC (1 μg/ml) for 30 min prior to analysis on a FACSCalibur.

### Glycan microarray analyses

Lipid-linked oligosaccharide probes (Table 1) were printed on nitrocellulose-coated glass slides (FAST slides, Whatman) in a dose–response format from 1 to 5 fmol/spot with a noncontact arrayer (Piezorray, PerkinElmer), including Cy3 dye in the array fluid to enable postarray monitoring of the spots [21]. These were in the form of synthetic glycolipids [22] and neoglycolipids (NGLs) derived from natural oligosaccharides [23]. Binding analysis was performed essentially as described previously [24]. In brief, the arrayed slides were overlaid initially for 1 h with blocking solution (1% [w/v] bovine serum albumin [BSA, Sigma] in 2% [v/v] Pierce Casein Blocker solution, 10 mM Tris–HCl [pH 8.0], and 150 mM NaCl) at ambient temperature. The slides were rinsed with blocking buffer and overlaid for 1.5 h with the biotinylated siglec–GFP constructs that had been precomplexed for 1 h at 4 °C with AlexaFluor-647–streptavidin (Molecular Probes) (1:1, w/w) and rabbit anti-GFP (Invitrogen) (1:6, w/w). This precomplexing condition was selected after trials of different ratios of reactants. The precomplexed proteins were applied onto the slides at a final concentration of approximately 1.5 μg/ml in blocking solution. Incubation was for 90 min at ambient temperature, and binding was detected by incubating with biotinylated anti-rabbit IgG (Sigma) (1:200, v/v) in blocking solution for 1 h followed by AlexaFluor-647-labeled streptavidin (1 μg/ml) in blocking solution for 35 min. Slides were scanned using a GenePix 4200AL scanner (Molecular Devices, Sunnyvale, CA, USA), and binding signals were quantified using GenePix Pro 6.0 (Molecular Devices). Data analysis and presentation were carried out using Microsoft Excel. The results are presented as means of fluorescence intensities of duplicate spots after background subtraction. The error bars represent half of the difference between the two values.

### Heparin saccharide microarray fabrication and interrogation

Saccharides from porcine mucosal heparin (Celsus Labs, Cincinnati, OH, USA) were generated via partial digestion with heparinase I (IBEX Technologies, Montreal, Canada) and size exclusion chromatography fractionation using the procedure described previously [25,26]. A concentration series of approximately 8mer saccharides was prepared, lyophilized, and resuspended in formamide (Sigma–Aldrich Chemie, Steinheim, Germany) supplemented with 1.5 M betaine (Sigma–Aldrich, St. Louis, MO, USA). Saccharide microarrays were fabricated onto GAPS II microarray slides (Corning, Corning, NY, USA) from low to high concentration with spots 0.36 mm apart using a single MicroSpot 2500 split pin and a MicroGrid TAS robot (Genomic Solutions, Huntingdon, UK) operated at 57% humidity and 17 °C. The coupling chemistry involves nucleophilic attack of the amine on gamma aminopropylsilane (GAPS) slides to the carbonyl of the heparin saccharide [27]. Spots were quality controlled visually under a microscope, and slides were immediately microwave heated (5 min at 50% power in an 850-W oven followed by 10 min cooling at room temperature in the dark) three times in succession [27]. Slides were washed with deionized water (∼pH 7.0), dried using an air compressor, and stored with dessicant at room temperature in a sealed bag until required.

Slides were blocked with 1% (w/v) BSA (Fraction V, Sigma–Aldrich) in PBS. Arrays were then sequentially incubated with approximately 20 nM biotinylated protein–EGFP conjugates in filtered physiological buffer, 1% BSA, 0.1% Tween 20 (Sigma–Aldrich), and 1 μg/ml AlexaFluor-546-labeled streptavidin (Invitrogen) in filtered PBS, 1% BSA, and 0.05% Tween 20. Incubations were performed for 1 h at room temperature using a 16-well array incubation chamber (Whatman) in a moist environment. Slides were washed with deionized water (∼pH 7.0) for 5 min after each incubation and dried using an air compressor.

Microarrays were scanned at 532 nm with 10 μm resolution using a GenePix 4000A scanner (Molecular Devices) operated with GenePix Pro 3.0 software. The photomultiplier tube intensity was adjusted to the maximum permitted without saturation of any signals. Fluorescent intensity analysis with GenePix Pro 3.0 software used features fitted by eye to spots so that the diameter matched that of the spot as closely as possible. Intensity values were exported into Excel, and the mean of the difference between the mean feature and background intensities for replicate spots was calculated.

### Assessment of protein–protein interactions using Biacore

Measurement of protein–protein interactions was performed by surface plasmon resonance using a Biacore X (Biacore, Uppsala, Sweden). Streptavidin-coated chips were obtained from Biacore. All experiments were performed in PBS containing 0.005% Tween 20 at pH 7.0. HCl (100 mM) was used for regeneration of the chips. To immobilize the proteins, biotin–JAM-B–EGFP or biotin–JAM-C–EGFP (8 μg/ml) was injected for 50 min over the surface of a streptavidin chip at a flow rate of 1 μl/min. Unbound protein was removed from the chip by washing with buffer. A JAM/anti-GFP Ab mixture (1:10 molar ratio) was prepared, incubated at room temperature for 1 h to allow complex formation, and then injected for 12 min over the surface of the JAM-B- or JAM-C-coated chip. The solution was replaced with running buffer for 6 min, and the chip was regenerated with HCl and used sequentially for the next injections of JAM/anti-GFP Ab. Anti-GFP Ab only (the amount used in the 1:10 JAM/anti-GFP Ab complex) was injected over the chip surface as a control at a flow rate of 5 μl/min. BIAevaluation software was used for analysis of the data.

## Results

### High-throughput cloning and expression of type 1 membrane and secreted proteins

**Fig.1:**
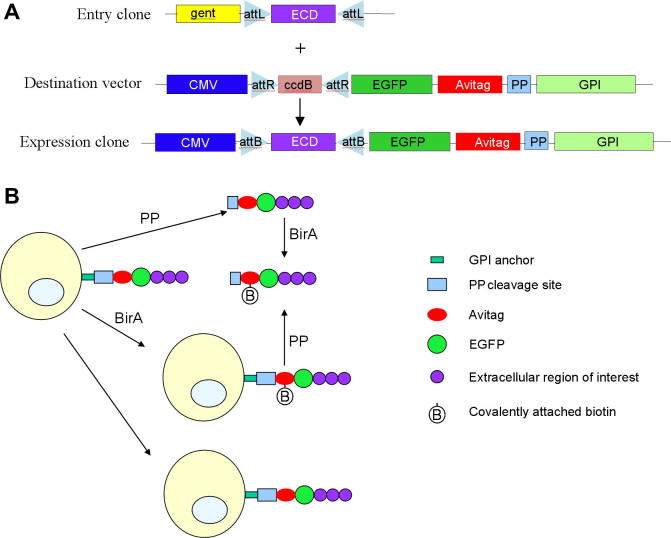
Gateway-based cloning and expression system. (A) An entry clone containing the leader peptide and extracellular domain (ECD) of interest is recombined via the LR reaction with a destination vector to generate an expression clone. This incorporates the ECD fused to EGFP, an Avitag biotin acceptor peptide, a PP cleavage site, and a GPI signal sequence. (B) Following expression in mammalian cells, surface-presented protein can be cleaved with PP and biotinylated with BirA either at the cell surface or following cleavage. This versatile cloning vector enables multiple assays with either cell-expressed or soluble versions of the protein of interest.

The Gateway cloning system has several advantages over conventional cloning strategies, particularly in terms of speed, simplicity, and versatility [16]. Fig. 1A illustrates the key features of the system developed for high-throughput surface expression and screening of type 1 membrane and secreted proteins for protein–glycan and protein–protein interactions. An entry clone containing the leader peptide and extracellular region of interest, corresponding to either a type 1 membrane protein or a secreted protein, is recombined with a destination vector to generate an expression clone. This encodes a GPI-anchored version of the protein of interest together with an EGFP tag, an Avitag biotin acceptor peptide, and a site for PP cleavage. This versatile expression system can be used to generate cell-bound and cleaved forms of proteins that can be optionally biotinylated using the BirA enzyme (Fig. 1B).

**Fig. 2:**
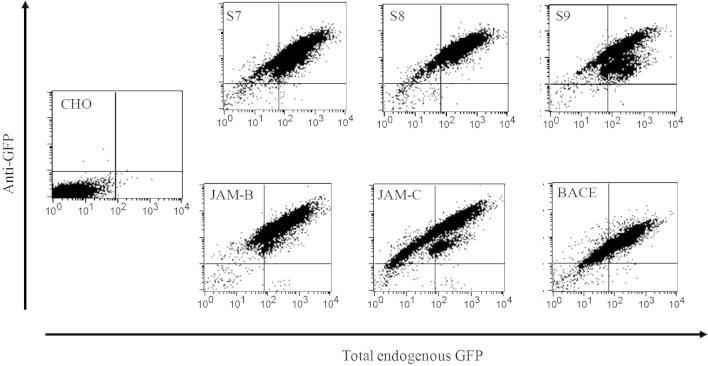
Expression of EGFP fusion proteins in CHO cells measured by flow cytometry. Cell surface expression of EGFP fusion proteins was measured on living cells using biotinylated anti-GFP followed by streptavidin–APC and detected on the FL-4 channel (emission maximum 660 nm). Total endogenous GFP was detected on the FL-1 channel (emission maximum 509 nm). CHO cells were either nontransfected (CHO) or CHO cells expressing siglec-7 (S7), siglec-8 (S8), siglec-9 (S9), BACE, JAM-B, or JAM-C.

To evaluate this system, we selected several well-characterized membrane and secreted proteins known to mediate either glycan or protein interactions. These included members of the siglec family of sialic acid-binding Ig-like lectins, the heparan sulfate-binding proteins FGF-1 and BACE, and the heterotypic adhesion molecule partners JAM-B and JAM-C. These proteins bind their ligands in a divalent cation-independent manner; therefore, binding assays were performed using Ca^2+^- and Mg^2+^-free media. Following drug selection and FACS sorting for EGFP expression, stably expressing CHO cell lines could be generated for all constructs tested. Flow cytometric analysis for total endogenous EGFP expression and surface-expressed EGFP revealed diagonal scatter in dot plots, indicating efficient surface expression (Fig. 2).

### Cell-based screens for protein–glycan interactions

**Fig.3:**
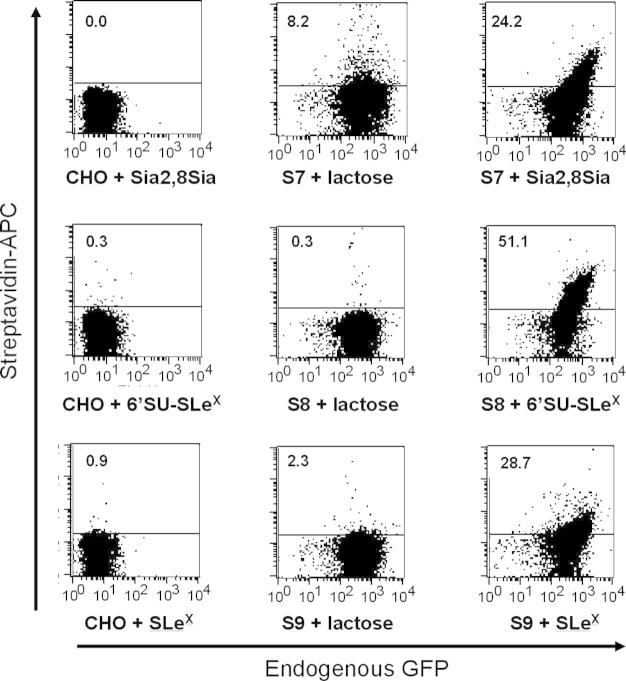
Binding of polymeric glycan probes to siglec-expressing CHO cells. Either nontransfected control cells (CHO) or CHO cells expressing siglec-7 (S7), siglec-8 (S8), or siglec-9 (S9) were incubated with biotinylated PAA probes carrying either lactose, Sia2,8Sia, SLe^x^, or 6′SU-SLe^x^ and binding detected with streptavidin–APC. See Table 1 for carbohydrate sequences.

To investigate whether the surface-expressed proteins could be used to measure protein–glycan interactions, we focussed on siglecs-7, -8, and -9, which are known from previous studies to have distinct preferences for sialylated glycans [24]. Clustered siglecs bind with high avidity to glycans presented in multimeric arrays on PAA backbones [19,28], but when siglec-binding activity is expressed at the cell surface, it is often inhibited by *cis* interactions with sialic acids [5]. Therefore, CHO cells expressing siglecs-7, -8, and -9 were treated with sialidase to remove the *cis*-interacting sialic acids and assayed for binding to PAA–glycans (Fig. 3 and Table 1). None of the siglec-expressing CHO cells bound to lactose–PAA used as a negative control, but selective binding was seen with PAA–glycans known to bind each siglec, namely Sia2,8Sia (siglec-7), 6′SU-SLe^x^ (siglec-8), and SLe^x^ (siglec-9) (Fig. 3 and Table 1). These results demonstrate the utility of the cell expression system for screening glycan interactions of membrane proteins.

### Cleavage of surface-expressed proteins

**Fig.4:**
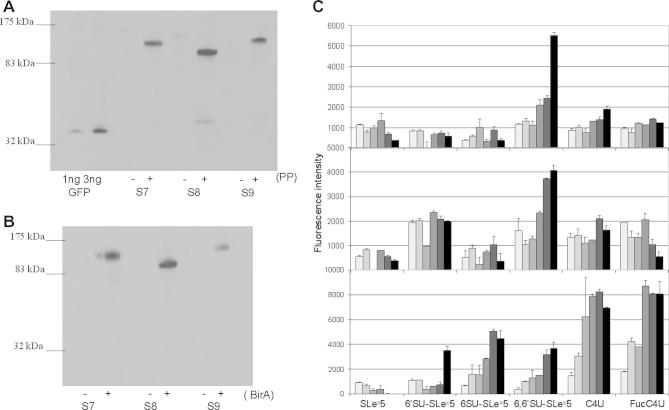
Preparation of soluble siglecs and protein–glycan interactions measured on glycan arrays. (A) Western blot of soluble material released from siglec-expressing CHO cells either treated (+) or not treated (−) with PP. Supernatants containing cleaved siglec-7–EGFP (S7), siglec-8–EGFP (S8), and siglec-9–EGFP (S9) were probed with biotinylated goat anti-GFP followed by streptavidin–alkaline phosphatase. (B) Western blot of cleaved material either pretreated (+) or not pretreated (−) with BirA enzyme and probed with streptavidin–alkaline phosphatase. (C) Dose–response microarray analysis of the binding of siglecs to lipid-linked oligosaccharide probes. Microarrays of six oligosaccharide probes (structures are shown in Table 1) were generated on nitrocellulose-coated glass slides. Each probe was printed in duplicate at 1 (), 1.33 (), 1.66 (), 2.33 (), 3.33 (), and 5 () fmol/spot. Binding of biotinylated siglec-7–EGFP (top panel), siglec-8–EGFP (middle panel), and siglec-9–EGFP (bottom panel) chimeras was detected with AlexaFluor-647-labeled streptavidin as described in Materials and methods.

To convert the GPI-anchored proteins to soluble proteins, cell pellets expressing siglecs-7, -8, and -9 were treated with PP under varying conditions and the amount of residual protein on the cell surface was measured by flow cytometry. PP cleaves between Gln and Gly residues of the recognition sequence Leu-Glu-Val-Leu-Phe-Gln-Gly-Pro [17], which was engineered into the destination vector. PP was able to cleave up to 90% of cell surface-expressed proteins within 4 h of incubation at 4 °C (data not shown). Cell viability was routinely greater than 90% under these conditions. The soluble protein released into the supernatant was measured by Western blotting using purified recombinant GFP as a standard (Fig. 4A) or by enzyme-linked immunosorbent assay (ELISA) (data not shown). By both approaches, yields of proteins were usually between 100 and 300 ng/10^7^ cells. Following PP treatment, Western blots of soluble material probed with anti-GFP antibodies showed specific bands corresponding to the expected sizes of the cleaved proteins (Fig. 4A). Treatment of the soluble cleaved-off proteins with BirA resulted in biotin incorporation, as shown in Western blots probed with streptavidin conjugate (Fig. 4B).

### Protein–glycan binding interactions

Glycan arrays provide a powerful miniaturized system to screen binding specificity of putative glycan-binding proteins against hundreds of different glycan structures [9,29]. In this validation study, we selected six lipid-linked sialylated glycans shown previously to be differentially recognized by siglecs-7, -8, and -9 [24]. To demonstrate binding of the soluble cleaved siglecs to glycan arrays, it was necessary to increase their avidities by precomplexing in solution, because monomeric siglecs exhibit very weak affinities, usually in the high-micromolar to low-millimolar range [30–32]. This was achieved by mixing biotinylated siglec–EGFP proteins with rabbit anti-GFP and streptavidin–AlexaFluor-647. As shown in Fig. 4C, each siglec fusion protein gave the predicted binding pattern that has been reported previously using recombinant siglecs fused to the Fc region of human IgG_1_ [24].

**Fig.5:**
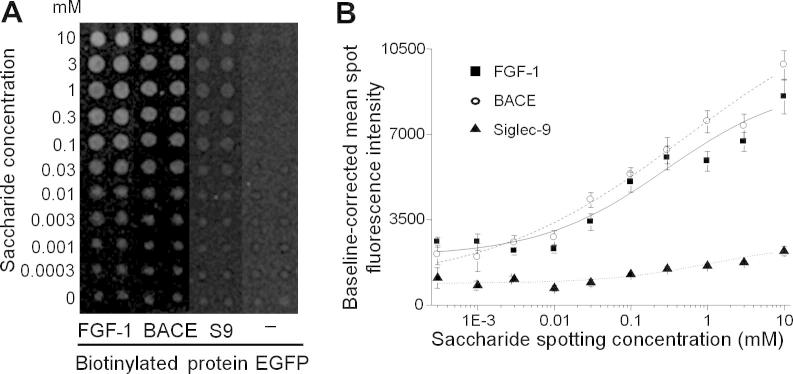
Measurement of protein–glycosaminoglycan interactions. Here 8mer saccharides derived from a partial heparinase I digestion of porcine mucosal heparin were spotted at different concentrations onto GAPS II microarray slides and incubated sequentially with 20 nM biotinylated protein–EGFP followed by AlexaFluor-546-labeled streptavidin. (A) Microarray image showing duplicate spots representative of 10 replicate spots. Contrast and brightness has been adjusted to show spot signal with respect to equivalent background signal. S9, siglec-9; −, streptavidin detection reagent applied to subarrays where no protein was incubated. (B) Graphs showing mean spot intensities against saccharide spotting concentration for different proteins. Spot intensity was normalized relative to background intensity by subtraction of the local background for each spot and the mean standard deviation of these values calculated for 10 replicate spots.

To investigate the potential for measuring interactions of proteins with glycosaminoglycans, we generated soluble biotinylated BACE–EGFP and FGF-1–EGFP fusion proteins and measured binding to immobilized heparan sulfate 8mer saccharides from porcine mucosal heparin (Fig. 5). Because of the relatively high affinity of BACE and FGF-1 for heparin [33,34], it was not necessary to precomplex the proteins prior to incubating with the arrays. Both biotin–BACE–EGFP and biotin–FGF-1–EGFP fusion proteins showed a saccharide dose-dependent increase in binding, whereas biotinylated siglec-9–EGFP served as negative control and exhibited negligible binding over the same concentration range. This pattern of binding of the recombinant proteins was comparable to that seen previously using commercially available FGF-1 (data not shown) binding to heparan sulfate saccharide microarrays [35].

### Protein–protein binding interactions

Having established the utility of the expression system to measure protein–glycan interactions, we next asked whether we could also measure weak protein–protein interactions. As a model system, we focused on JAM-B and JAM-C, which are thought to be a heterotypic pair of type 1 membrane cell adhesion molecules expressed on endothelial cells and some leukocytes [36]. JAMs are localized at intercellular contacts, where they participate in the assembly and maintenance of junctions, signaling to cytoskeleton-associated proteins, and recruiting cell polarity proteins to the junctions [37]. Three approaches were used to analyze interactions between JAM-B and JAM-C: cell-to-cell, protein-to-cell, and protein-to-protein binding assays.

**Fig.6:**
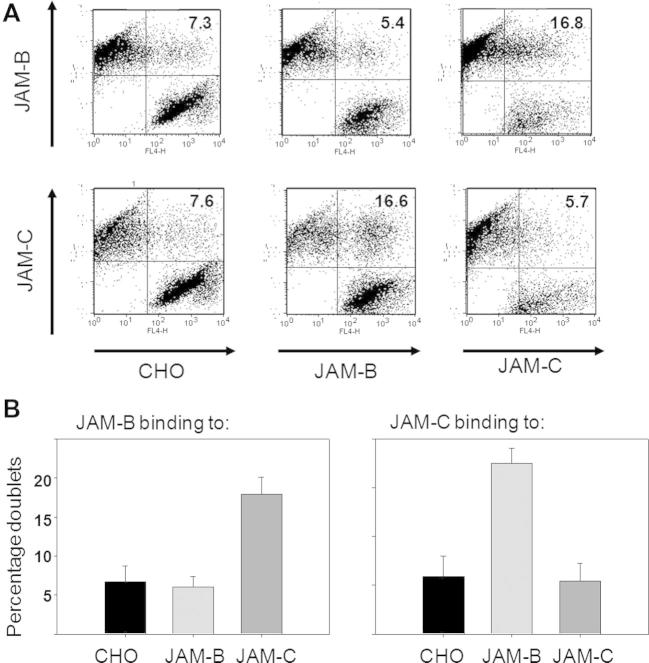
Measurement of protein–protein interactions via cell–cell binding assays. (A) CHO cells, either nontransfected (CHO) or expressing JAM-B or JAM-C, were labeled with either DiI or DiD molecular dye and mixed together in a 1:1 ratio. After incubation on ice for 60 min, the formation of doublets containing one cell of each type was evaluated by flow cytometry. (B) Results pooled from five experiments. Data represent means ± standard errors.

**Fig.7:**
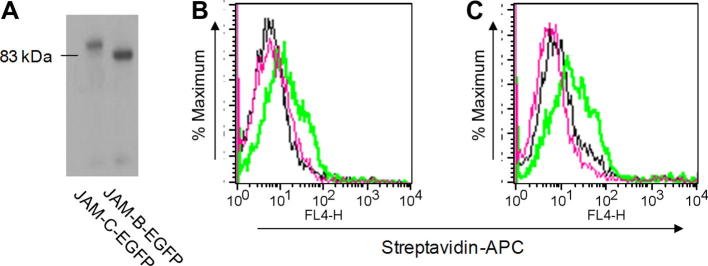
Binding of biotinylated JAM-B–EGFP and JAM-C–EGFP chimeras to CHO cells expressing JAM-B and JAM-C. (A) Western blot of biotinylated JAM-C or JAM-B probed with streptavidin–alkaline phosphatase shows the presence of a single species for each protein at the expected molecular weight. (B) Wild-type CHO cells (black line) or CHO cells expressing JAM-B (red and green lines) were incubated with either biotinylated JAM-C–EGFP (black and green lines) or biotinylated JAM-B–EGFP (red line) at 1 μg/ml. Binding was detected with streptavidin–APC, and cells were analyzed by flow cytometry. (C) Wild-type CHO cells (black line) or CHO cells expressing JAM-C (red and green lines) were incubated with either biotinylated JAM-B–EGFP (black and green lines) or biotinylated JAM-C–EGFP (red line). Binding was detected with streptavidin–APC, and cells were analyzed by flow cytometry. (For interpretation of the references to color in this figure legend, the reader is referred to the Web version of this article.)

**Fig.8:**
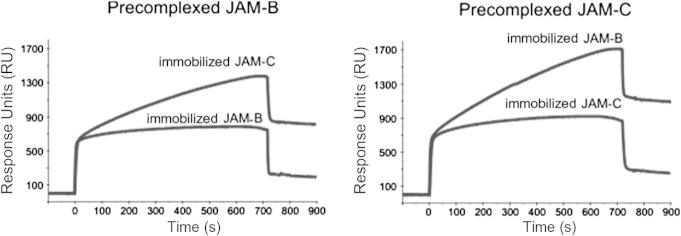
Analysis of interactions between JAM-B and JAM-C by surface plasmon resonance. Biotin–EGFP–JAM-B or biotin–EGFP–JAM-C was immobilized on streptavidin-coated Biacore chips. EGFP–JAM-B and EGFP–JAM-C were precomplexed with anti-GFP Ab and passed over the Biacore chips for 12 min before switching to buffer alone.

For cell-to-cell binding assays, nontransfected CHO cells or CHO cells expressing either JAM-B or JAM-C were fluorescently labeled with either DiI (red) or DiD (far red) molecular dye, and after mixing in a 1:1 ratio and pelleting, the formation of mixed doublets was assessed by flow cytometry (Fig. 6). This clearly demonstrated that CHO cells expressing either JAM-B or JAM-C formed mostly mixed cell doublets rather than cell doublets expressing only JAM-B or JAM-C (Fig. 6). The binding specificity was further demonstrated in protein-to-cell binding assays, in which biotinylated JAM-B–EGFP or JAM-C–EGFP fusion proteins (Fig. 7A) were precomplexed with streptavidin–APC and incubated with CHO cells expressing either protein and binding measured by flow cytometry (Fig. 7B, C). Finally, we explored the use of soluble cleaved proteins in protein-to-protein binding assays using surface plasmon resonance (Fig. 8). Biacore streptavidin chips were derivatized with biotin–JAM-B–EGFP or biotin–JAM-C–EGFP and probed with the same proteins that had been precomplexed with anti-GFP Ab. The sensorgrams show a sharp increase in response units at the beginning of the injection period and a decrease at the end that represents a background effect due to the difference in the refractive indexes of the running and sample buffers (data not shown). A continued slow increase in binding was seen only when precomplexed JAM-B interacted with immobilized JAM-C and vice versa (Fig. 8). At the end of the injection period, this was followed by a gradual decline, indicating slow dissociation of JAM-B/JAM-C complexes. These results clearly show the selective binding of JAM-B to JAM-C and vice versa.

## Discussion

A major interest of our group is to understand the biological roles of protein–glycan interactions. To date, there has been no systematic attempt to define the full repertoire of glycan-binding proteins at a proteomic scale in mammals. The purpose of the current study was to design a high-throughput cloning and expression system that was tailored around this goal, with a focus on membrane and secreted proteins. It was felt at the outset that having both cell-expressed and soluble forms of proteins would be important to maximize the chances of detecting very weak interactions that depend on clustering to achieve the high avidity required for stable binding. In the case of soluble proteins, the stoichiometry and spatial organization of multimeric complexes might not always be permissive to the formation of stable interactions with clustered ligands, whereas the same protein expressed at the cell surface could achieve this. For example, previous work showed that a cell-expressed form of the SIGN-R1 lectin can bind its polyvalent ligand dextran, whereas a recombinant soluble protein cannot do so even after precomplexing in solution [38].

To express proteins at the cell surface in a form suitable for multivalent glycan binding, our approach was to employ a GPI anchor that was fused at the C-terminal position of extracellular regions of interest. GPI-anchored proteins are efficiently transported from the Golgi to the outer leaflet of the plasma membrane, where they can undergo lateral diffusion and ligand-induced clustering [13]. GPI anchors can also direct proteins to cholesterol- and sphingolipid-enriched plasma membrane microdomains (lipid rafts), where they are also clustered. In this study, we used the GPI anchor signal sequence from the TRAIL-R3 protein, which has been shown to target reporter molecules to non-raft microdomains [12]. Thus, a common GPI anchor could direct a range of diverse proteins to the cell surface, making them available for ligand binding as well as for cleavage and conversion to soluble proteins.

The Gateway recombination system is well suited for high-throughput methodologies [16]. The destination vector we designed incorporates a number of features that both enable rapid selection of stably expressing cell lines and act as versatile tags to enable a range of binding assays. These include an EGFP tag that can be detected by its endogenous fluorescence or with specific Abs, a biotinylation motif, a PP cleavage site for optional generation of soluble proteins, and a GPI anchor signal sequence to direct efficient surface expression. Essentially, the destination vector allows expression of any peptide sequence containing its own leader peptide such as type 1 membrane proteins and secreted proteins. In the future, the destination vector could be modified to include a leader peptide that would allow expression and screening of other proteins lacking N-terminal leader peptides, including type II membrane proteins such as C-type lectins in which the reversed topology of the protein would still permit interactions with ligands [39]. A major feature of the current system is that both cell-expressed and soluble versions of the protein of interest can be generated from a single vector. This greatly reduces the amount of work required to generate each form, and although yields of soluble protein are relatively small compared with dedicated expression platforms, they are more than adequate for microscale screening of the types described here, including glycan arrays, flow cytometry, and surface plasmon resonance.

The methodologies established here lend themselves very favorably to large-scale genome-wide screening of membrane and secreted proteins for glycan interactions. By using the completed mammalian gene collection or commercial cDNAs as templates, preparation of hundreds of membrane and secreted proteins could be readily achieved [6]. Cell-expressed proteins could be screened either by using existing libraries of PAA–glycans [28] or by overlaying cells directly onto glycan arrays (unpublished observations). Using soluble proteins, the method of choice would be the glycan arrays with hundreds of immobilized probes, and the PAA libraries could be analyzed with immobilized soluble proteins. The same set of proteins could also be analyzed for protein–protein interactions. This could be achieved by analyzing cellular interactions with other cells or soluble proteins by flow cytometry, as demonstrated here, or by using cleaved soluble proteins. Here we demonstrated specific molecular interactions between JAM-B and JAM-C by surface plasmon resonance, but high-throughput screening of protein–protein interactions could be performed using protein microarrays that require only small amounts of material [40]. One advantage of the current system over recently described methods for analyzing protein–protein interaction networks of extracellular proteins [41] is that both cell-based and soluble protein-based methods can be used, thereby reducing the chances of missing important protein recognition due to protein misfolding or steric hindrance.

In conclusion, the combination of high-throughput protein cloning and expression technology combined with glycan and protein array technology provides new opportunities to discover novel molecular interactions of membrane and secreted proteins.

## Figures and Tables

**Table 1:** Glycan composition of probes.

^a^Polyacrylamide conjugates, 30 kDa, with approximately 40 glycan residues and 10 biotin residues per macromolecule. ^b^Glycolipids with a ceramide having 36 carbon atoms [22]. ^c^Neoglycolipids [23] prepared from reducing oligosaccharides by reductive amination with the amino lipid, 1,2-dihexadecyl-*sn*-glycero-3-phosphoethanolamine [42].

## Appendix A Supplementary data

Supplementary material Primers for PCRs.

## Footnotes

4 *Abbreviations used:* Ig, immunoglobulin; siglec, sialic acid-binding Ig-like lectin; GPI, glycosylphosphatidylinositol; PP, PreScission protease; FGF-1, fibroblast growth factor 1; BACE, β-site APP-cleaving enzyme; JAM, junctional adhesion molecule; EGFP, enhanced green fluorescent protein; GFP, green fluorescent protein; PCR, polymerase chain reaction; cDNA, complementary DNA; Int, integrase; IHF, integration host factor; Xis, excisionase; CHO, Chinese hamster ovary; FCS, fetal calf serum; Ab, antibody; APC, allophycocyanin; PBS, phosphate-buffered saline; EDTA, ethylenediaminetetraacetic acid; SDS–PAGE, sodium dodecyl sulfate–polyacrylamide gel electrophoresis; PAA, polyacrylamide; NGL, neoglycolipid; BSA, bovine serum albumin; GAPS, gamma aminopropylsilane; ELISA, enzyme-linked immunosorbent assay.
