# Supplementary material for: An expression system for screening of proteins for glycan and protein interactions
Source: Anal Biochem. 2011 Apr 15;411(2):261–70. doi: 10.1016/j.ab.2010.12.036 (PMC3740237; doi:10.1016/j.ab.2010.12.036)
Supplement: Supplementary material — Primers for PCRs. [file mmc1.doc]

**Supplementary Material**

**Primers for PCR reactions**

TRAIL receptor 3:

5’-TGTTGTACAAGGCCACTGTGGAAACCCCA- and 5’‑CTTGCGGCCGCTCAAACAAACACAATCAG (restriction sites are underlined).

PreScission protease:

5‑’ggacgagctgtacaagtctggccttgaagtgcttttccagggcccttctggcgccactgtggaaaccc and 5’‑gggtttccacagtggcgccagaagggccctggaaaagcacttcaaggccagacttgtacagctcgtcc

Avitag peptide:

5’‑ggacgagctgtacaagggcggccttaacgacattttcgaagctcagaagattgaatggcacgaatctggccttgaagtgc and 5’‑gcacttcaaggccagattcgtgccattcaatcttctgagcttcgaaaatgtcgttaaggccgcccttgtacagctcgtcc

Linker (for Avitag peptide):

5’‑Cgagctgtacaagggctctggctctcctggctctcctggccttaacgacattttc and 5’‑Gaaaatgtcgttaaggccaggagagccaggagagccagagcccttgtacagctcg

Primers for entry clones:

The forward and reverse primers for Siglec-F (Genbank accession number NM_145581) were (5’-3’) ATGCGGTGGGCATGGCTGCTG and CGTTCCTCTACTGGTTTC, for Siglec-10 (AF310233) ATGCTACTGCCACTGCTGCTG and GAGTCCCTTCTTATCTGG; for Siglec-9 (AF135027) ATGCTGCTGCTGCTGCTG, which was also used as forward primer for Siglec-7 and Siglec-8, and GCTCTGCAGGGAGACGTTCAG. The reverse primers for Siglec-7 (AF170485) and Siglec-8 (AF310234) were TCCTGATACAGGCCTCAT and CTCATTCTGCAGGGAGAGGCT respectively. Each forward primer contained an attB1 site and Kozak’s consensus sequence (underlined) ggggacaagtttgtacaaaaaagcaggcttcgccacc and the reverse primer an attB2 site ggggaccactttgtacaagaaagctgggtt, which are merged to coding region for 6-7 amino acids to generate 58 (forward) and 48 base (reverse) primers respectively. Forward and reverse primers for Jam-C (BC028778) were atggcgaggagcccccaaggc and gcttatgttgagaacatc; Jam-B (BC0243357) atggcgctgagccggcggctg and agcaatgttcaaatcata; Fgf-1 (BC037601) atggctgaaggggagatcaca and gtcagaagacaccgggag; Bace (BC048189) atggccccagcgctgcactgg and ataggctatggtcataag.
